# Na_7_Mg_13_Nd(PO_4_)_12_

**DOI:** 10.1107/S1600536812017850

**Published:** 2012-05-16

**Authors:** Hasna Jerbi, Mourad Hidouri, Ben Amara Mongi

**Affiliations:** a Faculté des Sciences, 5019 Monastir, Tunisia

## Abstract

Investigations of the quasi-ternary system Na_3_PO_4_–Mg_3_(PO_4_)_2_–NdPO_4_ allowed us to obtain the new phosphate hepta­sodium trideca­magnesium neodymium dodeca­kis­phosphate, Na_7_Mg_13_Nd(PO_4_)_12_, by applying a flux method. The crystal structure is isotypic with that of the previously reported Na_7_Mg_13_ *Ln*(PO_4_)_12_ (*Ln* = Eu, La) compounds. It consists of a complex three-dimensional framework built up from an NdO_8_ polyhedron (*m* symmetry), an *M*O_6_ octa­hedron statistically occupied by *M* = Mg and Na, and eight MgO_*x*_ (*x* = 5, 6) polyhedra (four with site symmetry *m*), linked either directely by sharing corners, edges and faces, or by one of the eight unique PO_4_ tetra­hedra through common corners. Two of the PO_4_ tetra­hedra are statisticaly disordered over a mirror plane. The whole structure can be described as resutling from an assembly of two types of structural units, *viz* [Mg_4_ *M*P_4_O_22_]_∞_^2^ layers extending parallel to (100) and stacked along [100], and [Mg_4_NdP_4_O_36_]_∞_^1^ undulating chains running along the [010] direction. The six different Na^+^ cations (five with site symmetry *m* and one with 0.5 occupancy) are situated in six distinct cavities delimited by the framework. The structure was refined from data of a racemic twin.

## Related literature

For the synthesis and luminescent properties of new phosphates for optical devices, see: Ngee *et al.* (2009 ▶); Shinde *et al.* (2011 ▶). The title structure is isotypic with Na_7_Mg_13_ *Ln*(PO_4_)_12_ (*Ln* = La, Eu) (Jerbi *et al.*, 2010 ▶). For P—O distances in orthophosphates, see: Baur (1974 ▶). For Mg—O distances, see: Ben Amara *et al.* (1983 ▶); Jaulmes *et al.* (1997 ▶); Klevtsova *et al.* (1980 ▶), For Na—O distances, see: Donnay & Allmann (1970 ▶), and for Nd—O distances, see: Albrand *et al.* (1974 ▶).

## Experimental

### 

#### Crystal data

Na_7_Mg_13_Nd(PO_4_)_12_
*M* *_r_* = 1760.84
Orthorhombic, 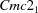

*a* = 10.301 (3) Å
*b* = 15.461 (4) Å
*c* = 23.384 (6) Å
*V* = 3724.2 (17) Å^3^
*Z* = 4
Mo *K*α radiation
μ = 2.38 mm^−1^
*T* = 293 K
0.40 × 0.22 × 0.17 mm

#### Data collection

Bruker–Nonius KappaCCD diffractometer
Absorption correction: analytical (Alcock, 1970 ▶) *T*_min_ = 0.716, *T*_max_ = 0.878
44522 measured reflections
11948 independent reflections
8244 reflections with *I* > 2σ(*I*)
*R*_int_ = 0.103

#### Refinement

*R*[*F* ^2^ > 2σ(*F* ^2^)] = 0.057
*wR*(*F* ^2^) = 0.107
*S* = 1.05
11948 reflections
424 parameters
1 restraint
Δρ_max_ = 2.37 e Å^−3^
Δρ_min_ = −2.23 e Å^−3^
Absolute structure: Flack (1983 ▶), 5847 Friedel pairs
Flack parameter: 0.257 (9)

### 

Data collection: *COLLECT* (Nonius, 1998 ▶); cell refinement: *SCALEPACK* (Otwinowski & Minor, 1997 ▶); data reduction: *SCALEPACK* and *DENZO* (Otwinowski & Minor, 1997 ▶); program(s) used to solve structure: *SIR92* (Altomare *et al.*, 1993 ▶); program(s) used to refine structure: *SHELXL97* (Sheldrick, 2008 ▶); molecular graphics: *DIAMOND* (Brandenburg, 1998 ▶); software used to prepare material for publication: *SHELXL97*.

## Supplementary Material

Crystal structure: contains datablock(s) global, I. DOI: 10.1107/S1600536812017850/wm2593sup1.cif

Structure factors: contains datablock(s) I. DOI: 10.1107/S1600536812017850/wm2593Isup2.hkl

Additional supplementary materials:  crystallographic information; 3D view; checkCIF report

## Figures and Tables

**Fig. 1.**
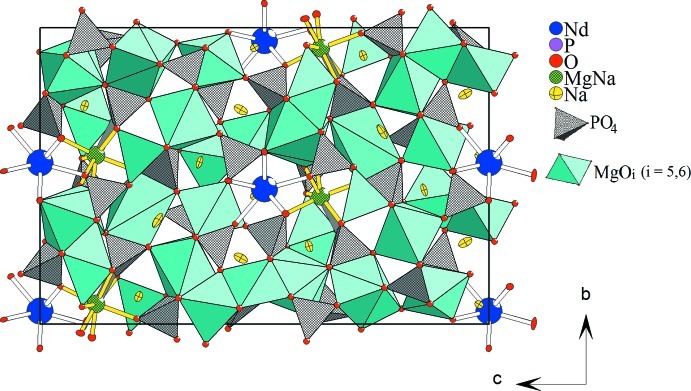
Projection of the structure along the [100] direction.

**Fig. 2.**
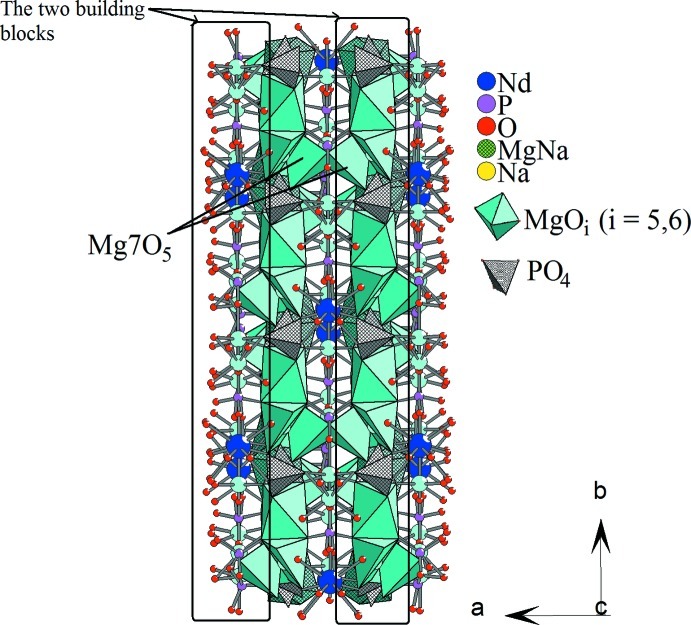
Projection along the [001] direction showing the [Mg4MP4O22]∞ layers and the [Mg4NdP4O36]∞ undulating chains.

**Fig. 3.**
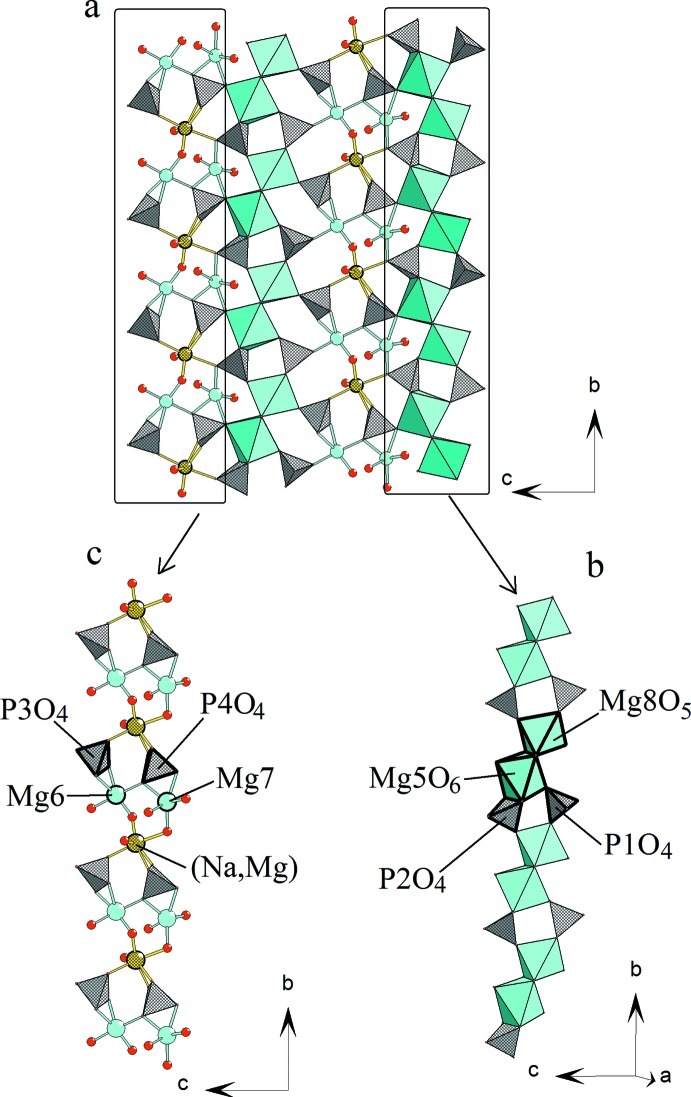
One [Mg4MP4O22]∞ layer

**Fig. 4.**
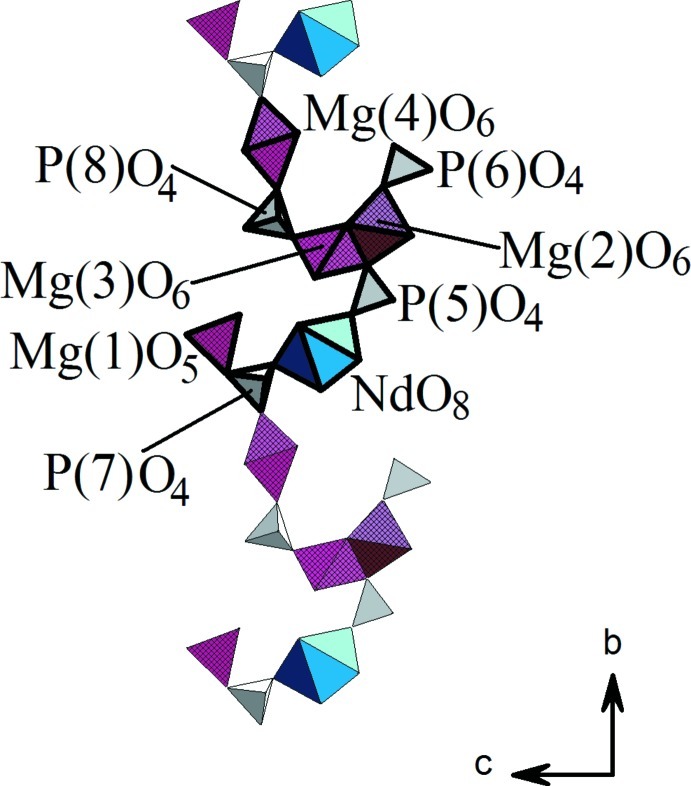
One [Mg4NdP4O36]∞ undulating chain

**Fig. 5.**
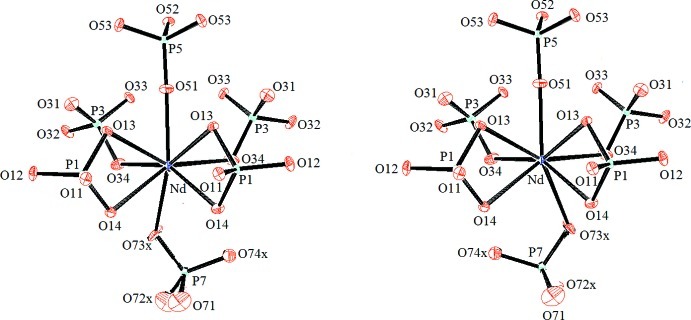
The left and right orientations of the (P7O4)x and (P8O4)x tetrahedra around the M site. Displacement ellipsoids are drawn at the 50% probability level.

## supplementary crystallographic information

### Comment

In recent years, the growing interest in new luminophores for light emitting diodes (LEDs) let to synthesis and characterization of news inorganic phosphates for use in next generation optical devices (Ngee *et al.*, 2009; Shinde *et al.* 2011). The knowledge of the crystal structure is necessary to understand the luminescence properties of the synthesized materials. Bearing this in mind, we recently started a systematic investigation of rare earth phosphates belonging to the quasi-ternary Na_3_PO_4_–Mg_3_(PO_4_)_2_–*Ln*PO_4_ (*Ln* is a lanthanoid) systems. As part of this ongoing study, the new phosphate Na_7_Mg_13_Nd(PO_4_)_12_ has been isolated in form of single crystals by applying a flux method. The underlying crystal structure is isotypic with the previously La and Eu analogues (Jerbi *et al.*, 2010).

The complex framework structure of the title compound is displayed in Fig. 1. It consists of an assembly of NdO_8_, MO_6_ (*M* = 0.5 Mg + 0.5 Na) and MgO*_x_* (*x* = 5, 6) polyhedra which are linked either directly through common corners, edges or faces, or by the phosphate groups. The Na^+^ cations are located within 6 crystallographically distinct cavities delimited by the framework.

As shown in Fig. 2, the whole structure can be decomposed into layers. [Mg_4_*M*P_4_O_22_]_∞_ layers extend parallel to (100) and are stacked along [100]. These layers are interconnected by [Mg_4_NdP_4_O_36_]_∞_ undulating chains which run along the [010] direction. The [Mg_4_*M*P_4_O_22_]_∞_ layer is made up of [Mg_2_P_2_O_12_]_∞_ chains alternating with [Mg_2_*M*P_2_O_14_]_∞_ ribbons, both spreading along the [010] direction. The [Mg_4_NdP_4_O_36_]_∞_ undulating chain (Fig. 4) is built from NdO_8_, Mg(1)O_5_ and Mg(4)O_6_ polyhedra and Mg_2_O_9_ bioctahedral units of face-sharing MgO_6_ octahedra. Such units are linked to each other by means of the PO_4_ tetrahedra through common corners. The connection of the layers and the undulating chains induces a rigid skeleton which forms six distinct cavities, occupied by the Na^+^ cations.

The environment of the Nd^3+^ site consists of eight O atoms with Nd—O distances in the range 2.385 (5) - 2.529 (3) Å and a mean distance of 2.499 Å, close to that of 2.484 Å, observed for the 8-coordinate Nd^3+^ in NdP_5_O_14_ (Albrand *et al.*, 1974). The *M* (*M* = 0.5 Mg + 0.5 Na) site is formally 8-coordinated. However, due to the statistically disordered phosphate tetrahedra (P7 with O73*x* and O74*x*; P8 with O83*x* and O(84)*x*), the effective number of O atoms surrounding the *M* is 6 (Fig. 5), and the average M—O distance is 2.175 Å. This is less than 2.230 Å as reported for the *M* (*M* = 0.5Mg + 0.5Na) site with a similar coordination in the molybdate Na_2_Mg_5_(MoO_4_)_6_ (Klevtsova *et al.*, 1980). The eight distinct Mg^2+^ sites display various coordination polyhedra. Mg2 to Mg5 are 6-coordinate with Mg—O distances in the range 1.961 (6) - 2.223 (4) Å leading to an overall distance of 2.097 Å, slightly lower but consistent with that of 2.14 Å as reported for octahedrally surrounded Mg^2+^ ions in Mg_3_(PO_4_)_2_ (Jaulmes *et al.*, 1997). Mg1, Mg6, Mg7 and Mg8 are 5-coordinate with Mg—O distances in the range 1.925 (9) - 2.348 (8) Å, leading to an overall value of 2.066 Å, close to that 2.080 Å reported for the likewise 5-coordinate Mg^2+^ ion in NaMg_4_(PO_4_)_3_ (Ben Amara *et al.*, 1983). The P—O distances within the PO_4_ tetrahedra are in the range 1.507 (6) - 1.563 (4) Å with an overall value of 1.533 Å, nearly the same of 1.537 Å as predicted by Baur for the orthophosphate group (Baur, 1974).

As already described above, the phosphate groups involving the atoms P7 and P8 are statistically disordered, occupying two positions which are slightly displaced from the (0,*y*,*z*) mirror plane. This leads to two orientations for each tetrahedron, namely a left and right orientation (Fig. 5). This behaviour is attributed to the need to accommodate the environments of neighboring *Ln* and *M* sites. For describing the environments of the Na^+^ cations Na—O distances below *L*_max_ = 3.13 Å, as suggested by Donnay and Allmann (1970) were taken into account. Hence their coordination numbers range from 5 to 10 with Na—O distances scattering from 1.968 (9) for the statistically disordered Na site to 2.979 (6) Å.

### Experimental

Single crystals of the Na_7_Mg_13_Nd(PO_4_)_12_ phoshate were grown in a flux of sodium molybdate Na_2_MoO_4_ starting from mixtures of Na_2_CO_3_, MgCO_3_, Nd_2_O_3_, NH_4_H_2_PO_4_ and Na_2_MoO_4_^.^2H_2_O in a molar ratio of 2.5:14:0.5:12:6. This mixture was ground in an agate mortar and heated in a platinum crucible for 24 h at 673 K to expel H_2_O, CO_2_ and NH_3_. After being reground, the product was molten for 2 h at 1273 K and subsequently cooled down to 773 K with a 10Kh^-1^ rate, after which the furnace was turned off. Hexagonally shaped crystals were obtained by washing the solidified product with warm water. Elemental analysis by ICP indicated the exclusive presence of Na, Nd, Mg and P in an atomic ratio of Na: Mg: Nd: P = 7.4:13.2: 0.95:12.

### Refinement

During refinement it turned out that each of the P7, P8, O72, O73, O74, O82, O83, O84 and Na6 atoms is statistically disordered over two positions (left and right), symmetrically related by the (0,*y*,*z*) mirror plane. These positions cannot be filled simultaneously. The MO_6_ site is likewise disordered and occupied statistically by Mg and Na atoms. The remaining highest and lowest electron densities are 2.37 e.Å^-3^ 0.36 Å from O84*x* and -2.33 e.Å^-3^ 0.03 Å from Nd, respectively. The crystal under investigation was an inversion twin with an approximate ratio of the twin domains of 3:1.

### Crystal data

**Table:**

Na_7_Mg_13_Nd(PO_4_)_12_	*D*_x_ = 3.140 Mg m^−^^3^
*M**_r_* = 1760.84	Melting point: 1007 K
Orthorhombic, *C**m**c*2_1_	Mo *K*α radiation, λ = 0.71069 Å
Hall symbol: C 2c -2	Cell parameters from 44599 reflections
*a* = 10.301 (3) Å	θ = 2.4–40°
*b* = 15.461 (4) Å	µ = 2.38 mm^−^^1^
*c* = 23.384 (6) Å	*T* = 293 K
*V* = 3724.2 (17) Å^3^	Parallelepiped, purple
*Z* = 4	0.40 × 0.22 × 0.17 mm
*F*(000) = 3428	

### Data collection

**Table:**

Bruker–Nonius KappaCCD diffractometer	11948 independent reflections
Radiation source: fine-focus sealed tube	8244 reflections with *I* > 2σ(*I*)
Graphite monochromator	*R*_int_ = 0.103
non–profiled ω scans	θ_max_ = 40.0°, θ_min_ = 3.2°
Absorption correction: analytical (Alcock, 1970)	*h* = −12→18
*T*_min_ = 0.716, *T*_max_ = 0.878	*k* = −27→27
44522 measured reflections	*l* = −42→42

### Refinement

**Table:**

Refinement on *F*^2^	Primary atom site location: structure-invariant direct methods
Least-squares matrix: full	Secondary atom site location: difference Fourier map
*R*[*F*^2^ > 2σ(*F*^2^)] = 0.057	*w* = 1/[σ^2^(*F*_o_^2^) + (0.0124*P*)^2^ + 42.9558*P*] where *P* = (*F*_o_^2^ + 2*F*_c_^2^)/3
*wR*(*F*^2^) = 0.107	(Δ/σ)_max_ = 0.001
*S* = 1.05	Δρ_max_ = 2.37 e Å^−^^3^
11948 reflections	Δρ_min_ = −2.23 e Å^−^^3^
424 parameters	Absolute structure: Flack (1983), 5847 Friedel pairs
1 restraint	Flack parameter: 0.257 (9)

### Special details

**Table:**

Geometry. All e.s.d.'s (except the e.s.d. in the dihedral angle between two l.s. planes) are estimated using the full covariance matrix. The cell e.s.d.'s are taken into account individually in the estimation of e.s.d.'s in distances, angles and torsion angles; correlations between e.s.d.'s in cell parameters are only used when they are defined by crystal symmetry. An approximate (isotropic) treatment of cell e.s.d.'s is used for estimating e.s.d.'s involving l.s. planes.
Refinement. Refinement of *F*^2^ against ALL reflections. The weighted *R*-factor *wR* and goodness of fit *S* are based on *F*^2^, conventional *R*-factors *R* are based on *F*, with *F* set to zero for negative *F*^2^. The threshold expression of *F*^2^ > σ(*F*^2^) is used only for calculating *R*-factors(gt) *etc*. and is not relevant to the choice of reflections for refinement. *R*-factors based on *F*^2^ are statistically about twice as large as those based on *F*, and *R*- factors based on ALL data will be even larger.

### Fractional atomic coordinates and isotropic or equivalent isotropic displacement parameters (Å^2^)

**Table:**

	*x*	*y*	*z*	*U*_iso_*/*U*_eq_	Occ. (<1)
Nd	0.0000	0.54408 (2)	0.000015 (13)	0.00904 (5)	
P1	0.21976 (9)	0.43115 (7)	0.05989 (4)	0.00612 (16)	
O11	0.1824 (3)	0.3576 (2)	0.09995 (13)	0.0116 (6)	
O12	0.3671 (3)	0.4462 (2)	0.05621 (14)	0.0129 (6)	
O13	0.1558 (3)	0.41806 (18)	0.00077 (17)	0.0125 (5)	
O14	0.1526 (3)	0.5162 (2)	0.08102 (14)	0.0112 (5)	
P2	0.74694 (10)	0.46442 (7)	0.22216 (5)	0.00978 (18)	
O21	0.7715 (4)	0.5517 (2)	0.19519 (16)	0.0232 (8)	
O22	0.7106 (3)	0.4816 (2)	0.28494 (15)	0.0191 (7)	
O23	0.6391 (3)	0.4113 (2)	0.19233 (14)	0.0125 (6)	
O24	0.8668 (3)	0.4038 (2)	0.21746 (13)	0.0107 (5)	
P3	0.24602 (11)	0.70154 (7)	0.98592 (4)	0.00913 (18)	
O31	0.2995 (3)	0.6688 (2)	0.04252 (14)	0.0168 (7)	
O32	0.1777 (4)	0.6272 (2)	0.95352 (15)	0.0170 (7)	
O33	0.3605 (3)	0.7387 (2)	0.95155 (16)	0.0153 (6)	
O34	0.1511 (3)	0.7784 (2)	0.98766 (14)	0.0160 (7)	
P4	0.23934 (11)	0.72710 (8)	0.81954 (5)	0.0116 (2)	
O41	0.3634 (4)	0.6820 (3)	0.8390 (2)	0.0337 (12)	
O42	0.2489 (5)	0.7603 (3)	0.75815 (16)	0.0286 (9)	
O43	0.1310 (3)	0.6600 (2)	0.82915 (14)	0.0143 (6)	
O44	0.2043 (4)	0.8076 (2)	0.85537 (15)	0.0210 (8)	
P5	0.0000	0.72322 (10)	0.11586 (7)	0.0069 (3)	
O51	0.0000	0.6619 (3)	0.0654 (2)	0.0144 (8)	
O52	0.0000	0.8177 (3)	0.09809 (19)	0.0105 (8)	
O53	0.1213 (3)	0.7066 (2)	0.15281 (15)	0.0132 (6)	
P6	0.5000	0.64818 (10)	0.16991 (6)	0.0060 (2)	
O61	0.6206 (3)	0.7003 (2)	0.15437 (17)	0.0180 (7)	
O62	0.5000	0.5682 (3)	0.1316 (2)	0.0294 (14)	
O63	0.5000	0.6231 (4)	0.2330 (2)	0.0169 (10)	
P7	0.02504 (19)	0.55827 (15)	0.36368 (10)	0.0078 (5)	0.50
O71	0.0000	0.5300 (3)	0.3018 (2)	0.0166 (9)	
O72X	−0.020 (3)	0.6490 (5)	0.3742 (4)	0.037 (6)	0.50
O73X	0.0621 (7)	0.4980 (6)	0.3999 (3)	0.0252 (18)	0.50
O74X	0.1652 (7)	0.5446 (5)	0.3848 (3)	0.0177 (12)*	0.50
P8	0.5147 (8)	0.53079 (15)	0.39974 (9)	0.0120 (12)	0.50
O81	0.5000	0.5645 (4)	0.3388 (2)	0.0255 (13)	
O82X	0.4470 (6)	0.5929 (4)	0.4417 (3)	0.0130 (12)	0.50
O83X	0.3373 (6)	0.5306 (4)	0.4107 (3)	0.0141 (12)	0.50
O84X	0.5429 (8)	0.4411 (6)	0.4069 (4)	0.037 (2)*	0.50
Mg1	0.0000	0.40172 (15)	0.28024 (9)	0.0100 (4)	
Mg2	0.5000	0.43819 (13)	0.13099 (9)	0.0072 (4)	
Mg3	0.0000	0.85120 (14)	0.01707 (9)	0.0082 (4)	
Mg4	0.0000	0.78245 (17)	0.39476 (10)	0.0115 (4)	
Mg5	0.76130 (15)	0.29805 (10)	0.17575 (7)	0.0091 (3)	
Mg6	0.2319 (3)	0.63904 (12)	0.43096 (8)	0.0284 (5)	
Mg7	0.34561 (18)	0.60958 (11)	0.28670 (8)	0.0187 (4)	
Mg8	0.24440 (18)	0.61726 (10)	0.11851 (7)	0.0155 (3)	
Mg	0.2460 (2)	0.56877 (13)	0.87518 (8)	0.0254 (5)	0.50
Na	0.2460 (2)	0.56877 (13)	0.87518 (8)	0.0254 (5)	0.50
Na1	0.0000	0.0937 (2)	0.78091 (13)	0.0195 (6)	
Na2	0.0000	0.2634 (2)	0.40137 (14)	0.0226 (7)	
Na3	0.0000	0.4531 (3)	0.14512 (14)	0.0235 (8)	
Na4	0.5000	0.4330 (2)	0.52265 (14)	0.0230 (6)	
Na5	0.5000	0.7765 (2)	0.05347 (18)	0.0352 (9)	
Na6	0.9685 (5)	0.6495 (4)	0.2386 (2)	0.0455 (19)	0.50

### Atomic displacement parameters (Å^2^)

**Table:**

	*U*^11^	*U*^22^	*U*^33^	*U*^12^	*U*^13^	*U*^23^
Nd	0.00955 (11)	0.00944 (11)	0.00815 (11)	0.000	0.000	0.00003 (13)
P1	0.0045 (4)	0.0058 (4)	0.0080 (4)	−0.0004 (3)	−0.0007 (3)	0.0007 (3)
O11	0.0116 (14)	0.0103 (14)	0.0129 (14)	−0.0008 (11)	−0.0013 (11)	0.0024 (11)
O12	0.0077 (12)	0.0131 (15)	0.0180 (14)	0.0004 (10)	−0.0045 (11)	0.0040 (12)
O13	0.0140 (12)	0.0133 (12)	0.0101 (11)	0.0056 (9)	−0.0042 (14)	−0.0014 (15)
O14	0.0107 (13)	0.0078 (12)	0.0152 (14)	0.0017 (10)	0.0009 (11)	−0.0026 (11)
P2	0.0077 (4)	0.0110 (5)	0.0107 (4)	0.0020 (4)	0.0012 (3)	0.0015 (4)
O21	0.032 (2)	0.0138 (16)	0.0238 (18)	−0.0055 (15)	−0.0075 (15)	0.0097 (14)
O22	0.0187 (16)	0.0227 (18)	0.0158 (16)	0.0043 (13)	0.0081 (13)	−0.0005 (14)
O23	0.0083 (13)	0.0147 (15)	0.0146 (15)	0.0024 (11)	0.0001 (10)	0.0006 (12)
O24	0.0075 (12)	0.0174 (14)	0.0073 (12)	0.0037 (11)	0.0006 (10)	0.0014 (11)
P3	0.0102 (4)	0.0082 (4)	0.0090 (4)	−0.0014 (3)	0.0035 (4)	0.0002 (3)
O31	0.0216 (17)	0.0185 (16)	0.0102 (14)	−0.0027 (13)	0.0001 (12)	0.0042 (12)
O32	0.0230 (17)	0.0127 (15)	0.0152 (15)	−0.0051 (13)	−0.0003 (13)	−0.0008 (12)
O33	0.0140 (14)	0.0133 (15)	0.0187 (15)	0.0007 (12)	0.0105 (12)	0.0022 (12)
O34	0.0102 (13)	0.0164 (15)	0.0213 (17)	0.0026 (11)	0.0038 (11)	−0.0065 (12)
P4	0.0090 (4)	0.0146 (5)	0.0113 (5)	0.0012 (4)	−0.0023 (4)	−0.0070 (4)
O41	0.0142 (16)	0.044 (3)	0.043 (2)	0.0165 (17)	−0.0161 (17)	−0.036 (2)
O42	0.041 (2)	0.031 (2)	0.0138 (18)	−0.0208 (19)	0.0055 (17)	−0.0077 (15)
O43	0.0123 (13)	0.0217 (17)	0.0088 (13)	−0.0002 (12)	−0.0021 (11)	−0.0017 (12)
O44	0.028 (2)	0.0195 (18)	0.0151 (16)	0.0066 (15)	−0.0036 (14)	−0.0102 (13)
P5	0.0079 (6)	0.0051 (6)	0.0076 (6)	0.000	0.000	0.0011 (5)
O51	0.021 (2)	0.0100 (19)	0.012 (2)	0.000	0.000	−0.0024 (16)
O52	0.015 (2)	0.0055 (18)	0.0110 (19)	0.000	0.000	0.0003 (15)
O53	0.0095 (14)	0.0115 (15)	0.0185 (15)	−0.0013 (11)	−0.0040 (11)	0.0006 (12)
P6	0.0053 (6)	0.0062 (6)	0.0065 (6)	0.000	0.000	0.0004 (5)
O61	0.0110 (15)	0.0175 (17)	0.0255 (19)	−0.0056 (12)	−0.0036 (13)	0.0066 (14)
O62	0.071 (5)	0.006 (2)	0.012 (2)	0.000	0.000	−0.0005 (18)
O63	0.019 (2)	0.023 (3)	0.009 (2)	0.000	0.000	0.0034 (18)
P7	0.0061 (12)	0.0098 (9)	0.0075 (8)	0.0002 (6)	0.0002 (6)	−0.0024 (7)
O71	0.027 (3)	0.014 (2)	0.0083 (19)	0.000	0.000	−0.0022 (16)
O72X	0.051 (18)	0.012 (3)	0.047 (5)	0.011 (5)	0.000 (5)	−0.012 (3)
O73X	0.019 (3)	0.049 (5)	0.008 (3)	0.021 (3)	0.001 (2)	0.007 (3)
P8	0.012 (4)	0.0146 (9)	0.0094 (7)	−0.0055 (11)	0.0032 (9)	−0.0058 (6)
O81	0.021 (3)	0.049 (4)	0.007 (2)	0.000	0.000	−0.007 (2)
O82X	0.014 (3)	0.018 (3)	0.007 (3)	−0.001 (2)	−0.004 (2)	−0.006 (2)
O83X	0.009 (3)	0.011 (3)	0.023 (3)	−0.004 (2)	0.007 (2)	−0.003 (2)
Mg1	0.0107 (9)	0.0119 (10)	0.0072 (9)	0.000	0.000	0.0004 (8)
Mg2	0.0089 (9)	0.0069 (9)	0.0057 (8)	0.000	0.000	−0.0012 (7)
Mg3	0.0084 (9)	0.0100 (9)	0.0063 (8)	0.000	0.000	0.0006 (7)
Mg4	0.0047 (8)	0.0212 (12)	0.0085 (9)	0.000	0.000	0.0029 (8)
Mg5	0.0094 (6)	0.0081 (6)	0.0097 (6)	0.0007 (5)	0.0006 (5)	0.0002 (5)
Mg6	0.0638 (15)	0.0095 (8)	0.0119 (8)	0.0020 (9)	0.0194 (9)	0.0014 (6)
Mg7	0.0232 (9)	0.0122 (8)	0.0207 (9)	−0.0036 (6)	0.0076 (7)	−0.0063 (7)
Mg8	0.0256 (8)	0.0089 (7)	0.0121 (7)	−0.0053 (6)	0.0074 (6)	−0.0026 (6)
Mg	0.0535 (14)	0.0087 (8)	0.0140 (8)	0.0107 (8)	−0.0124 (9)	−0.0020 (7)
Na	0.0535 (14)	0.0087 (8)	0.0140 (8)	0.0107 (8)	−0.0124 (9)	−0.0020 (7)
Na1	0.0121 (12)	0.0292 (17)	0.0172 (14)	0.000	0.000	−0.0063 (13)
Na2	0.0154 (14)	0.0356 (19)	0.0167 (15)	0.000	0.000	0.0073 (14)
Na3	0.0152 (15)	0.043 (2)	0.0125 (14)	0.000	0.000	0.0112 (14)
Na4	0.0312 (17)	0.0170 (14)	0.0208 (15)	0.000	0.000	−0.0035 (12)
Na5	0.0243 (17)	0.0295 (19)	0.052 (2)	0.000	0.000	−0.0176 (17)
Na6	0.047 (5)	0.057 (3)	0.032 (3)	0.009 (3)	0.000 (2)	0.029 (3)

### Geometric parameters (Å, º)

**Table:**

Nd—O51	2.379 (5)	P8—Mg6^vii^	3.185 (7)
Nd—O32^i^	2.487 (4)	P8—Na^v^	3.219 (7)
Nd—O32^ii^	2.487 (4)	O81—P8^vii^	1.525 (6)
Nd—O14^iii^	2.499 (3)	O81—Mg7	2.121 (4)
Nd—O14	2.499 (3)	O81—Mg7^vii^	2.121 (4)
Nd—O73X^iv^	2.513 (7)	O81—Na1^x^	2.796 (7)
Nd—O73X^v^	2.513 (7)	O82X—O82X^vii^	1.091 (13)
Nd—O13^iii^	2.525 (3)	O82X—P8^vii^	1.428 (7)
Nd—O13	2.525 (3)	O82X—O83X	1.652 (9)
Nd—P1^iii^	3.1834 (12)	O82X—Mg3^xiii^	2.037 (6)
Nd—P1	3.1834 (12)	O82X—Mg6	2.342 (7)
Nd—Na3	3.673 (3)	O82X—Na2^xvi^	2.853 (8)
P1—O11	1.523 (3)	O83X—P8^vii^	1.546 (10)
P1—O12	1.538 (3)	O83X—Na^v^	1.984 (7)
P1—O13	1.545 (4)	O83X—Mg^v^	1.984 (7)
P1—O14	1.565 (3)	O83X—Mg6	2.052 (7)
P1—Na3	3.035 (3)	O84X—O84X^vii^	0.885 (17)
P1—Mg8	3.197 (2)	O84X—P8^vii^	1.517 (10)
P1—Na5^vi^	3.296 (3)	O84X—Na^ix^	2.303 (9)
O11—Mg5^vii^	2.079 (3)	O84X—Mg^ix^	2.303 (9)
O11—Na5^vi^	2.507 (4)	O84X—Mg4^viii^	2.509 (10)
O11—Na3	2.613 (4)	O84X—Na4	2.745 (10)
O12—Mg3^viii^	2.206 (4)	Mg1—O24^xviii^	2.010 (3)
O12—Mg2	2.224 (3)	Mg1—O24^vii^	2.010 (3)
O12—Na4^ix^	2.445 (4)	Mg1—O43^iv^	2.010 (4)
O13—Mg6^v^	2.015 (4)	Mg1—O43^v^	2.010 (4)
O13—Na5^vi^	2.981 (5)	Mg1—P2^xviii^	3.0952 (17)
O14—Mg8	2.026 (3)	Mg1—P2^vii^	3.0952 (17)
O14—Na3	2.381 (4)	Mg1—P7^iii^	3.119 (3)
P2—O21	1.511 (4)	Mg1—Na3	3.258 (4)
P2—O22	1.538 (4)	Mg1—Na^v^	3.400 (3)
P2—O23	1.547 (4)	Mg1—Mg^v^	3.400 (3)
P2—O24	1.554 (3)	Mg2—O52^viii^	2.015 (5)
P2—Mg5	2.796 (2)	Mg2—O23^vii^	2.070 (4)
P2—Mg7^vii^	2.868 (2)	Mg2—O12^vii^	2.224 (3)
P2—Na1^x^	3.027 (2)	Mg2—Mg3^viii^	2.984 (3)
P2—Mg1^xi^	3.0952 (17)	Mg2—Na4^ix^	3.222 (4)
P2—Na3^xi^	3.174 (2)	Mg2—Na1^x^	3.540 (4)
O21—Mg8^vii^	2.066 (4)	Mg2—Mg5^vii^	3.610 (2)
O21—Mg7^vii^	2.614 (5)	Mg2—Mg5	3.610 (2)
O21—Na6	2.727 (7)	Mg3—O82X^xxi^	2.037 (6)
O22—Mg7^vii^	2.062 (4)	Mg3—O82X^xxii^	2.037 (6)
O22—Na^ix^	2.293 (4)	Mg3—O34^i^	2.040 (3)
O22—Mg^ix^	2.293 (4)	Mg3—O34^ii^	2.040 (3)
O22—Na1^x^	2.464 (4)	Mg3—O12^xxiii^	2.206 (4)
O23—Mg2	2.070 (4)	Mg3—O12^xix^	2.206 (4)
O23—Mg5	2.191 (4)	Mg3—Mg2^xix^	2.984 (3)
O23—Na1^x^	2.520 (4)	Mg3—Na2^iv^	3.234 (4)
O24—Mg1^xi^	2.010 (3)	Mg3—Na4^xxii^	3.338 (4)
O24—Mg5	2.192 (4)	Mg3—Mg6^xxi^	3.421 (3)
O24—Na3^xi^	2.308 (4)	Mg4—O33^xxi^	1.984 (4)
P3—O31^xii^	1.521 (3)	Mg4—O33^xxii^	1.984 (4)
P3—O33	1.538 (3)	Mg4—O41^xxi^	1.995 (4)
P3—O34	1.540 (3)	Mg4—O41^xxii^	1.995 (4)
P3—O32	1.546 (4)	Mg4—O72X^iii^	2.129 (8)
P3—Mg6^xiii^	2.789 (2)	Mg4—O84X^xix^	2.509 (10)
P3—Na2^xiv^	3.260 (2)	Mg4—O84X^xxiii^	2.509 (10)
P3—Na5^xii^	3.269 (3)	Mg4—Na1^iv^	3.279 (4)
P3—Na4^xv^	3.451 (2)	Mg4—Mg6^iii^	3.368 (3)
O31—P3^ii^	1.521 (3)	Mg4—Mg6	3.368 (3)
O31—Mg8	2.029 (4)	Mg4—Mg^xxii^	3.514 (3)
O31—Na4^ix^	2.638 (4)	Mg5—O61^xxiv^	2.004 (4)
O31—Na5	2.665 (4)	Mg5—O11^vii^	2.079 (3)
O32—Mg	2.160 (4)	Mg5—O53^viii^	2.089 (4)
O32—Nd^xii^	2.487 (4)	Mg5—O42^ix^	2.130 (4)
O32—Na2^xiv^	2.775 (5)	Mg5—Mg8^viii^	3.104 (2)
O33—Mg4^xiii^	1.984 (4)	Mg5—Na3^xi^	3.508 (3)
O33—Mg6^xiii^	2.171 (4)	Mg5—Na6^xxiv^	3.612 (5)
O33—Na5^xii^	2.844 (5)	Mg5—Na5^viii^	3.786 (4)
O34—Mg3^xii^	2.040 (3)	Mg6—O13^xxv^	2.015 (4)
O34—Mg6^xiii^	2.200 (4)	Mg6—O44^xxii^	2.058 (4)
O34—Na2^xiv^	2.629 (4)	Mg6—O33^xxii^	2.171 (4)
P4—O41	1.525 (4)	Mg6—O34^xxii^	2.200 (4)
P4—O42	1.528 (4)	Mg6—O72X^iii^	2.56 (2)
P4—O43	1.540 (4)	Mg6—P3^xxii^	2.789 (2)
P4—O44	1.543 (4)	Mg6—P8^vii^	3.185 (7)
P4—Mg	2.773 (2)	Mg7—O22^vii^	2.062 (4)
P4—Mg7^xiii^	2.780 (2)	Mg7—O44^xxii^	2.117 (4)
P4—Na2^xiv^	3.124 (2)	Mg7—O42^xxii^	2.332 (5)
O41—Mg4^xiii^	1.995 (4)	Mg7—O21^vii^	2.614 (5)
O41—Mg	2.289 (6)	Mg7—P4^xxii^	2.780 (2)
O41—Na1^xvi^	2.386 (5)	Mg7—P2^vii^	2.868 (2)
O42—Mg5^xv^	2.130 (4)	Mg7—Mg7^vii^	3.181 (4)
O42—Mg7^xiii^	2.332 (5)	Mg7—Na6^vii^	3.481 (6)
O42—Na6^xvii^	2.696 (7)	Mg7—Na1^x^	3.525 (4)
O43—Mg1^xiv^	2.010 (4)	Mg7—Mg^v^	3.597 (3)
O43—Mg	2.133 (4)	Mg8—O21^vii^	2.066 (4)
O43—Na2^xiv^	2.465 (4)	Mg8—O61^vii^	2.070 (4)
O44—Mg6^xiii^	2.058 (4)	Mg8—Mg5^xix^	3.104 (2)
O44—Mg7^xiii^	2.117 (4)	Mg8—Na4^ix^	3.544 (3)
O44—Na2^xiv^	2.606 (5)	Mg8—Na6^vii^	3.598 (6)
P5—O51	1.513 (5)	Mg—O74X^xxv^	1.954 (8)
P5—O52	1.519 (5)	Mg—O83X^xxv^	1.984 (7)
P5—O53^iii^	1.541 (3)	Mg—O73X^xxv^	2.233 (7)
P5—O53	1.541 (3)	Mg—O22^xv^	2.293 (4)
P5—Mg8^iii^	3.004 (2)	Mg—O84X^xv^	2.303 (9)
P5—Mg8	3.004 (2)	Mg—P8^xv^	2.962 (7)
P5—Mg3	3.042 (3)	Mg—P7^xxv^	3.018 (3)
P5—Na6^vii^	3.106 (5)	Mg—Mg1^xiv^	3.400 (3)
P5—Na6^xviii^	3.106 (5)	Na1—O41^xxvi^	2.386 (5)
O52—Mg3	1.964 (5)	Na1—O41^vi^	2.386 (5)
O52—Mg2^xix^	2.015 (5)	Na1—O22^xxvii^	2.464 (4)
O53—Mg8	2.040 (4)	Na1—O22^xxviii^	2.464 (4)
O53—Mg5^xix^	2.089 (4)	Na1—O23^xxvii^	2.520 (4)
O53—Na6^vii^	2.380 (6)	Na1—O23^xxviii^	2.520 (4)
O53—Na6^xviii^	2.699 (6)	Na1—O81^xxvii^	2.796 (7)
P6—O61^vii^	1.524 (4)	Na1—P2^xxvii^	3.027 (2)
P6—O61	1.524 (4)	Na1—P2^xxviii^	3.027 (2)
P6—O62	1.526 (5)	Na1—Mg4^xiv^	3.279 (4)
P6—O63	1.526 (5)	Na1—P8^xxviii^	3.384 (4)
P6—Mg8	2.934 (2)	Na1—P8^xxvii^	3.384 (4)
P6—Mg8^vii^	2.934 (2)	Na2—O43^iv^	2.465 (4)
P6—Na5	3.369 (5)	Na2—O43^v^	2.465 (4)
O61—Mg5^xx^	2.004 (4)	Na2—O44^iv^	2.606 (5)
O61—Mg8^vii^	2.070 (4)	Na2—O44^v^	2.606 (5)
O61—Na5	2.915 (6)	Na2—O34^iv^	2.629 (4)
O62—Mg2	2.011 (6)	Na2—O34^v^	2.629 (4)
O62—Na4^ix^	2.548 (6)	Na2—O32^v^	2.775 (5)
O62—Mg8	2.757 (3)	Na2—O32^iv^	2.775 (5)
O62—Mg8^vii^	2.757 (3)	Na2—O82X^vi^	2.853 (8)
O63—Mg7^vii^	2.037 (4)	Na2—O82X^xxvi^	2.853 (8)
O63—Mg7	2.037 (4)	Na2—P4^iv^	3.124 (2)
P7—P7^iii^	0.516 (4)	Na2—P4^v^	3.124 (2)
P7—O73X	1.316 (8)	Na3—O24^vii^	2.308 (4)
P7—O72X^iii^	1.424 (7)	Na3—O24^xviii^	2.308 (4)
P7—O72X	1.497 (12)	Na3—O14^iii^	2.381 (4)
P7—O71	1.534 (5)	Na3—O11^iii^	2.613 (4)
P7—O74X	1.540 (7)	Na3—P1^iii^	3.035 (3)
P7—O73X^iii^	1.546 (7)	Na3—P2^vii^	3.174 (2)
P7—O74X^iii^	2.032 (7)	Na3—P2^xviii^	3.174 (2)
P7—Mg6	2.929 (3)	Na3—Na5^vi^	3.470 (5)
P7—Na^v^	3.018 (3)	Na4—O12^xv^	2.445 (4)
P7—Mg^v^	3.018 (3)	Na4—O12^xxv^	2.445 (4)
P7—Mg1	3.119 (3)	Na4—O62^xv^	2.548 (6)
O71—P7^iii^	1.534 (5)	Na4—O31^xxv^	2.638 (4)
O71—Mg1	2.046 (5)	Na4—O31^xv^	2.638 (4)
O71—Na6^xviii^	2.387 (7)	Na4—O84X^vii^	2.745 (10)
O71—Na6^vii^	2.387 (7)	Na4—Mg2^xv^	3.222 (4)
O72X—O72X^iii^	0.41 (6)	Na4—P8^vii^	3.251 (4)
O72X—P7^iii^	1.424 (7)	Na4—Na5^xv^	3.319 (5)
O72X—Mg4	2.129 (8)	Na4—Mg3^xiii^	3.338 (4)
O72X—Mg6^iii^	2.56 (2)	Na5—O11^xvi^	2.507 (4)
O73X—O73X^iii^	1.279 (14)	Na5—O11^xxiii^	2.507 (4)
O73X—O74X	1.332 (11)	Na5—O31^vii^	2.665 (4)
O73X—P7^iii^	1.546 (7)	Na5—O33^ii^	2.844 (5)
O73X—Na^v^	2.233 (7)	Na5—O33^xxix^	2.844 (5)
O73X—Mg^v^	2.233 (7)	Na5—O61^vii^	2.915 (6)
O73X—Nd^xiv^	2.513 (7)	Na5—O13^xxiii^	2.981 (5)
O74X—Mg6	1.941 (8)	Na5—O13^xvi^	2.981 (5)
O74X—Na^v^	1.954 (8)	Na5—P3^ii^	3.269 (3)
O74X—Mg^v^	1.954 (8)	Na5—P3^xxix^	3.269 (3)
O74X—P7^iii^	2.032 (7)	Na6—Na6^xxx^	0.649 (11)
P8—P8^vii^	0.304 (16)	Na6—O53^vii^	2.380 (6)
P8—O84X	1.426 (10)	Na6—O71^xi^	2.387 (7)
P8—O82X^vii^	1.428 (7)	Na6—O42^xxxi^	2.696 (7)
P8—O84X^vii^	1.517 (10)	Na6—O53^xi^	2.699 (6)
P8—O81	1.525 (6)	Na6—P5^xi^	3.106 (5)
P8—O82X	1.540 (7)	Na6—P7^vii^	3.247 (5)
P8—O83X^vii^	1.546 (10)	Na6—P7^xi^	3.298 (5)
P8—O83X	1.846 (10)	Na6—Mg7^vii^	3.481 (6)
P8—Na^ix^	2.962 (7)	Na6—Mg8^vii^	3.598 (6)
P8—Mg^ix^	2.962 (7)	Na6—Mg5^xx^	3.612 (5)
			
O51—Nd—O32^i^	83.43 (11)	O34^xxii^—Mg6—O72X^iii^	138.2 (2)
O51—Nd—O32^ii^	83.43 (11)	O82X—Mg6—O72X^iii^	151.8 (3)
O32^i^—Nd—O32^ii^	94.83 (17)	O63—Mg7—O22^vii^	107.8 (2)
O51—Nd—O14^iii^	69.20 (11)	O63—Mg7—O44^xxii^	126.5 (2)
O32^i^—Nd—O14^iii^	87.56 (11)	O22^vii^—Mg7—O44^xxii^	121.83 (17)
O32^ii^—Nd—O14^iii^	152.10 (10)	O63—Mg7—O81	78.59 (17)
O51—Nd—O14	69.20 (11)	O22^vii^—Mg7—O81	84.7 (2)
O32^i^—Nd—O14	152.10 (10)	O44^xxii^—Mg7—O81	86.87 (19)
O32^ii^—Nd—O14	87.56 (11)	O63—Mg7—O42^xxii^	93.50 (19)
O14^iii^—Nd—O14	77.97 (14)	O22^vii^—Mg7—O42^xxii^	134.79 (17)
O51—Nd—O73X^iv^	142.9 (2)	O44^xxii^—Mg7—O42^xxii^	66.04 (15)
O32^i^—Nd—O73X^iv^	62.54 (18)	O81—Mg7—O42^xxii^	139.5 (2)
O32^ii^—Nd—O73X^iv^	85.0 (2)	O63—Mg7—O21^vii^	83.75 (16)
O14^iii^—Nd—O73X^iv^	120.1 (2)	O22^vii^—Mg7—O21^vii^	61.68 (13)
O14—Nd—O73X^iv^	145.25 (18)	O44^xxii^—Mg7—O21^vii^	135.69 (17)
O51—Nd—O73X^v^	142.9 (2)	O81—Mg7—O21^vii^	134.72 (19)
O32^i^—Nd—O73X^v^	85.0 (2)	O42^xxii^—Mg7—O21^vii^	82.42 (14)
O32^ii^—Nd—O73X^v^	62.54 (18)	O14—Mg8—O31	93.14 (15)
O14^iii^—Nd—O73X^v^	145.25 (18)	O14—Mg8—O53	113.75 (15)
O14—Nd—O73X^v^	120.1 (2)	O31—Mg8—O53	104.63 (16)
O51—Nd—O13^iii^	125.91 (10)	O14—Mg8—O21^vii^	87.71 (16)
O32^i^—Nd—O13^iii^	86.19 (11)	O31—Mg8—O21^vii^	167.17 (18)
O32^ii^—Nd—O13^iii^	150.44 (11)	O53—Mg8—O21^vii^	86.66 (16)
O14^iii^—Nd—O13^iii^	57.41 (11)	O14—Mg8—O61^vii^	165.25 (16)
O14—Nd—O13^iii^	105.15 (11)	O31—Mg8—O61^vii^	85.58 (16)
O73X^iv^—Nd—O13^iii^	69.2 (2)	O53—Mg8—O61^vii^	80.71 (14)
O73X^v^—Nd—O13^iii^	88.21 (19)	O21^vii^—Mg8—O61^vii^	90.34 (17)
O51—Nd—O13	125.91 (10)	O14—Mg8—O62	106.35 (15)
O32^i^—Nd—O13	150.44 (11)	O31—Mg8—O62	86.43 (16)
O32^ii^—Nd—O13	86.19 (11)	O53—Mg8—O62	137.41 (16)
O14^iii^—Nd—O13	105.15 (11)	O21^vii^—Mg8—O62	81.06 (17)
O14—Nd—O13	57.41 (11)	O61^vii^—Mg8—O62	58.92 (15)
O73X^iv^—Nd—O13	88.21 (19)	O74X^xxv^—Mg—O83X^xxv^	57.2 (3)
O73X^v^—Nd—O13	69.2 (2)	O74X^xxv^—Mg—O43	114.5 (2)
O13^iii^—Nd—O13	78.96 (13)	O83X^xxv^—Mg—O43	170.6 (3)
O11—P1—O12	113.35 (18)	O74X^xxv^—Mg—O32	98.0 (2)
O11—P1—O13	110.18 (18)	O83X^xxv^—Mg—O32	97.0 (2)
O12—P1—O13	112.99 (18)	O43—Mg—O32	88.34 (15)
O11—P1—O14	108.76 (18)	O74X^xxv^—Mg—O73X^xxv^	36.3 (3)
O12—P1—O14	109.08 (18)	O83X^xxv^—Mg—O73X^xxv^	86.3 (3)
O13—P1—O14	101.78 (16)	O43—Mg—O73X^xxv^	88.0 (3)
O21—P2—O22	106.5 (2)	O32—Mg—O73X^xxv^	72.4 (2)
O21—P2—O23	113.9 (2)	O74X^xxv^—Mg—O41	162.5 (3)
O22—P2—O23	110.3 (2)	O83X^xxv^—Mg—O41	119.8 (2)
O21—P2—O24	112.1 (2)	O43—Mg—O41	66.51 (14)
O22—P2—O24	111.42 (19)	O32—Mg—O41	99.53 (16)
O23—P2—O24	102.61 (18)	O73X^xxv^—Mg—O41	153.8 (3)
O31^xii^—P3—O33	107.5 (2)	O74X^xxv^—Mg—O22^xv^	83.3 (2)
O31^xii^—P3—O34	117.7 (2)	O83X^xxv^—Mg—O22^xv^	91.7 (2)
O33—P3—O34	102.28 (19)	O43—Mg—O22^xv^	82.45 (14)
O31^xii^—P3—O32	110.1 (2)	O32—Mg—O22^xv^	170.34 (17)
O33—P3—O32	111.7 (2)	O73X^xxv^—Mg—O22^xv^	104.3 (2)
O34—P3—O32	107.3 (2)	O41—Mg—O22^xv^	79.46 (16)
O41—P4—O42	112.4 (3)	O74X^xxv^—Mg—O84X^xv^	107.8 (3)
O41—P4—O43	104.8 (2)	O83X^xxv^—Mg—O84X^xv^	50.7 (3)
O42—P4—O43	114.2 (2)	O43—Mg—O84X^xv^	137.0 (3)
O41—P4—O44	113.7 (2)	O32—Mg—O84X^xv^	93.5 (3)
O42—P4—O44	104.7 (2)	O73X^xxv^—Mg—O84X^xv^	133.4 (3)
O43—P4—O44	107.1 (2)	O41—Mg—O84X^xv^	70.8 (3)
O51—P5—O52	112.9 (3)	O22^xv^—Mg—O84X^xv^	95.2 (3)
O51—P5—O53^iii^	109.44 (18)	O41^xxvi^—Na1—O41^vi^	72.3 (2)
O52—P5—O53^iii^	108.27 (17)	O41^xxvi^—Na1—O22^xxvii^	140.18 (17)
O51—P5—O53	109.44 (18)	O41^vi^—Na1—O22^xxvii^	74.28 (14)
O52—P5—O53	108.27 (17)	O41^xxvi^—Na1—O22^xxviii^	74.28 (14)
O53^iii^—P5—O53	108.4 (3)	O41^vi^—Na1—O22^xxviii^	140.18 (17)
O61^vii^—P6—O61	109.1 (3)	O22^xxvii^—Na1—O22^xxviii^	123.4 (2)
O61^vii^—P6—O62	106.7 (2)	O41^xxvi^—Na1—O23^xxvii^	145.3 (2)
O61—P6—O62	106.7 (2)	O41^vi^—Na1—O23^xxvii^	98.65 (13)
O61^vii^—P6—O63	111.39 (19)	O22^xxvii^—Na1—O23^xxvii^	61.06 (12)
O61—P6—O63	111.39 (19)	O22^xxviii^—Na1—O23^xxvii^	121.17 (16)
O62—P6—O63	111.2 (3)	O41^xxvi^—Na1—O23^xxviii^	98.65 (14)
O73X—P7—O72X^iii^	126.7 (7)	O41^vi^—Na1—O23^xxviii^	145.3 (2)
O73X—P7—O72X	130.4 (5)	O22^xxvii^—Na1—O23^xxviii^	121.17 (16)
O73X—P7—O71	117.0 (4)	O22^xxviii^—Na1—O23^xxviii^	61.06 (12)
O72X^iii^—P7—O71	115.9 (5)	O23^xxvii^—Na1—O23^xxviii^	69.34 (17)
O72X—P7—O71	111.7 (5)	O41^xxvi^—Na1—O81^xxvii^	102.98 (19)
O72X^iii^—P7—O74X	96.5 (11)	O41^vi^—Na1—O81^xxvii^	102.98 (19)
O72X—P7—O74X	111.4 (10)	O22^xxvii^—Na1—O81^xxvii^	64.39 (11)
O71—P7—O74X	114.9 (3)	O22^xxviii^—Na1—O81^xxvii^	64.39 (11)
O72X^iii^—P7—O73X^iii^	118.5 (9)	O23^xxvii^—Na1—O81^xxvii^	111.76 (16)
O72X—P7—O73X^iii^	107.2 (9)	O23^xxviii^—Na1—O81^xxvii^	111.76 (16)
O71—P7—O73X^iii^	104.3 (3)	O43^iv^—Na2—O43^v^	66.40 (17)
O74X—P7—O73X^iii^	106.6 (4)	O43^iv^—Na2—O44^iv^	58.49 (12)
O73X—P7—O74X^iii^	92.9 (4)	O43^v^—Na2—O44^iv^	111.21 (16)
O72X^iii^—P7—O74X^iii^	91.4 (11)	O43^iv^—Na2—O44^v^	111.21 (16)
O72X—P7—O74X^iii^	76.0 (10)	O43^v^—Na2—O44^v^	58.49 (12)
O71—P7—O74X^iii^	92.1 (2)	O44^iv^—Na2—O44^v^	107.7 (2)
O74X—P7—O74X^iii^	144.3 (5)	O43^iv^—Na2—O34^iv^	108.65 (10)
O84X—P8—O82X^vii^	121.1 (5)	O43^v^—Na2—O34^iv^	165.5 (2)
P8^vii^—P8—O84X^vii^	66.9 (4)	O44^iv^—Na2—O34^iv^	74.65 (12)
O82X^vii^—P8—O84X^vii^	130.2 (5)	O44^v^—Na2—O34^iv^	133.75 (17)
O84X—P8—O81	117.6 (5)	O43^iv^—Na2—O34^v^	165.5 (2)
O82X^vii^—P8—O81	116.1 (4)	O43^v^—Na2—O34^v^	108.65 (10)
O84X^vii^—P8—O81	112.1 (5)	O44^iv^—Na2—O34^v^	133.75 (17)
O84X—P8—O82X	128.5 (5)	O44^v^—Na2—O34^v^	74.65 (12)
O84X^vii^—P8—O82X	108.8 (6)	O34^iv^—Na2—O34^v^	72.59 (16)
O81—P8—O82X	109.7 (4)	O43^iv^—Na2—O32^v^	111.67 (17)
O84X—P8—O83X^vii^	77.2 (5)	O43^v^—Na2—O32^v^	69.32 (12)
O82X^vii^—P8—O83X^vii^	67.4 (4)	O44^iv^—Na2—O32^v^	166.14 (17)
O84X^vii^—P8—O83X^vii^	111.5 (5)	O44^v^—Na2—O32^v^	84.56 (11)
O81—P8—O83X^vii^	104.7 (4)	O34^iv^—Na2—O32^v^	101.72 (15)
O82X—P8—O83X^vii^	110.0 (4)	O34^v^—Na2—O32^v^	54.69 (11)
O84X—P8—O83X	100.6 (5)	O43^iv^—Na2—O32^iv^	69.32 (12)
O82X^vii^—P8—O83X	100.3 (4)	O43^v^—Na2—O32^iv^	111.67 (17)
O84X^vii^—P8—O83X	66.1 (5)	O44^iv^—Na2—O32^iv^	84.56 (11)
O81—P8—O83X	91.8 (4)	O44^v^—Na2—O32^iv^	166.14 (17)
O82X—P8—O83X	57.6 (4)	O34^iv^—Na2—O32^iv^	54.69 (11)
O83X^vii^—P8—O83X	162.4 (5)	O34^v^—Na2—O32^iv^	101.72 (15)
O24^xviii^—Mg1—O24^vii^	86.12 (19)	O32^v^—Na2—O32^iv^	82.55 (18)
O24^xviii^—Mg1—O43^iv^	87.97 (13)	O43^iv^—Na2—O82X^vi^	124.47 (17)
O24^vii^—Mg1—O43^iv^	151.84 (19)	O43^v^—Na2—O82X^vi^	140.8 (2)
O24^xviii^—Mg1—O43^v^	151.84 (19)	O44^iv^—Na2—O82X^vi^	65.98 (16)
O24^vii^—Mg1—O43^v^	87.97 (13)	O44^v^—Na2—O82X^vi^	84.37 (18)
O43^iv^—Mg1—O43^v^	84.4 (2)	O34^iv^—Na2—O82X^vi^	53.55 (16)
O24^xviii^—Mg1—O71	99.48 (16)	O34^v^—Na2—O82X^vi^	68.42 (16)
O24^vii^—Mg1—O71	99.48 (16)	O32^v^—Na2—O82X^vi^	122.98 (17)
O43^iv^—Mg1—O71	108.65 (16)	O32^iv^—Na2—O82X^vi^	106.98 (16)
O43^v^—Mg1—O71	108.65 (16)	O43^iv^—Na2—O82X^xxvi^	140.8 (2)
O62—Mg2—O52^viii^	158.0 (2)	O43^v^—Na2—O82X^xxvi^	124.47 (17)
O62—Mg2—O23	101.27 (16)	O44^iv^—Na2—O82X^xxvi^	84.37 (18)
O52^viii^—Mg2—O23	94.54 (15)	O44^v^—Na2—O82X^xxvi^	65.98 (16)
O62—Mg2—O23^vii^	101.27 (16)	O34^iv^—Na2—O82X^xxvi^	68.42 (16)
O52^viii^—Mg2—O23^vii^	94.54 (15)	O34^v^—Na2—O82X^xxvi^	53.55 (16)
O23—Mg2—O23^vii^	87.7 (2)	O32^v^—Na2—O82X^xxvi^	106.98 (16)
O62—Mg2—O12	87.15 (16)	O32^iv^—Na2—O82X^xxvi^	122.98 (17)
O52^viii^—Mg2—O12	75.59 (14)	O82X^vi^—Na2—O82X^xxvi^	22.1 (3)
O23—Mg2—O12	169.17 (16)	O24^vii^—Na3—O24^xviii^	72.97 (18)
O23^vii^—Mg2—O12	97.46 (13)	O24^vii^—Na3—O14^iii^	171.6 (2)
O62—Mg2—O12^vii^	87.15 (16)	O24^xviii^—Na3—O14^iii^	101.77 (11)
O52^viii^—Mg2—O12^vii^	75.59 (14)	O24^vii^—Na3—O14	101.77 (11)
O23—Mg2—O12^vii^	97.46 (13)	O24^xviii^—Na3—O14	171.6 (2)
O23^vii^—Mg2—O12^vii^	169.17 (16)	O14^iii^—Na3—O14	82.65 (18)
O12—Mg2—O12^vii^	75.97 (18)	O24^vii^—Na3—O11^iii^	122.51 (19)
O52—Mg3—O82X^xxi^	161.1 (2)	O24^xviii^—Na3—O11^iii^	71.46 (12)
O52—Mg3—O82X^xxii^	161.1 (2)	O14^iii^—Na3—O11^iii^	60.14 (11)
O82X^xxi^—Mg3—O82X^xxii^	31.1 (4)	O14—Na3—O11^iii^	116.87 (17)
O52—Mg3—O34^i^	100.35 (15)	O24^vii^—Na3—O11	71.46 (12)
O82X^xxi^—Mg3—O34^i^	74.8 (2)	O24^xviii^—Na3—O11	122.51 (19)
O82X^xxii^—Mg3—O34^i^	98.4 (2)	O14^iii^—Na3—O11	116.87 (17)
O52—Mg3—O34^ii^	100.35 (15)	O14—Na3—O11	60.14 (11)
O82X^xxi^—Mg3—O34^ii^	98.4 (2)	O11^iii^—Na3—O11	91.98 (18)
O82X^xxii^—Mg3—O34^ii^	74.8 (2)	O12^xv^—Na4—O12^xxv^	68.10 (16)
O34^i^—Mg3—O34^ii^	99.4 (2)	O12^xv^—Na4—O62^xv^	71.64 (14)
O52—Mg3—O12^xxiii^	77.01 (14)	O12^xxv^—Na4—O62^xv^	71.64 (14)
O82X^xxi^—Mg3—O12^xxiii^	104.0 (2)	O12^xv^—Na4—O31^xxv^	146.90 (16)
O82X^xxii^—Mg3—O12^xxiii^	84.9 (2)	O12^xxv^—Na4—O31^xxv^	87.77 (11)
O34^i^—Mg3—O12^xxiii^	168.63 (15)	O62^xv^—Na4—O31^xxv^	79.58 (13)
O34^ii^—Mg3—O12^xxiii^	91.93 (13)	O12^xv^—Na4—O31^xv^	87.77 (11)
O52—Mg3—O12^xix^	77.01 (14)	O12^xxv^—Na4—O31^xv^	146.90 (16)
O82X^xxi^—Mg3—O12^xix^	84.9 (2)	O62^xv^—Na4—O31^xv^	79.58 (13)
O82X^xxii^—Mg3—O12^xix^	104.0 (2)	O31^xxv^—Na4—O31^xv^	103.03 (19)
O34^i^—Mg3—O12^xix^	91.93 (13)	O12^xv^—Na4—O84X	101.0 (2)
O34^ii^—Mg3—O12^xix^	168.63 (15)	O12^xxv^—Na4—O84X	111.8 (2)
O12^xxiii^—Mg3—O12^xix^	76.70 (17)	O62^xv^—Na4—O84X	170.47 (19)
O33^xxi^—Mg4—O33^xxii^	92.9 (2)	O31^xxv^—Na4—O84X	109.1 (2)
O33^xxi^—Mg4—O41^xxi^	88.36 (17)	O31^xv^—Na4—O84X	94.3 (2)
O33^xxii^—Mg4—O41^xxi^	173.5 (2)	O12^xv^—Na4—O84X^vii^	111.8 (2)
O33^xxi^—Mg4—O41^xxii^	173.5 (2)	O12^xxv^—Na4—O84X^vii^	101.0 (2)
O33^xxii^—Mg4—O41^xxii^	88.36 (17)	O62^xv^—Na4—O84X^vii^	170.47 (18)
O41^xxi^—Mg4—O41^xxii^	89.7 (3)	O31^xxv^—Na4—O84X^vii^	94.3 (2)
O33^xxi^—Mg4—O72X	85.5 (6)	O31^xv^—Na4—O84X^vii^	109.1 (2)
O33^xxii^—Mg4—O72X	93.5 (6)	O84X—Na4—O84X^vii^	18.5 (4)
O41^xxi^—Mg4—O72X	93.0 (6)	O11^xvi^—Na5—O11^xxiii^	97.14 (19)
O41^xxii^—Mg4—O72X	100.8 (6)	O11^xvi^—Na5—O31	159.2 (2)
O33^xxi^—Mg4—O72X^iii^	93.5 (6)	O11^xxiii^—Na5—O31	76.89 (11)
O33^xxii^—Mg4—O72X^iii^	85.5 (6)	O11^xvi^—Na5—O31^vii^	76.89 (11)
O41^xxi^—Mg4—O72X^iii^	100.8 (6)	O11^xxiii^—Na5—O31^vii^	159.2 (2)
O41^xxii^—Mg4—O72X^iii^	93.0 (6)	O31—Na5—O31^vii^	101.6 (2)
O72X—Mg4—O72X^iii^	11.1 (15)	O11^xvi^—Na5—O33^ii^	147.69 (19)
O33^xxi^—Mg4—O84X^xix^	102.3 (2)	O11^xxiii^—Na5—O33^ii^	95.00 (11)
O33^xxii^—Mg4—O84X^xix^	87.6 (2)	O31—Na5—O33^ii^	53.08 (11)
O41^xxi^—Mg4—O84X^xix^	85.9 (3)	O31^vii^—Na5—O33^ii^	100.52 (15)
O41^xxii^—Mg4—O84X^xix^	71.3 (3)	O11^xvi^—Na5—O33^xxix^	95.00 (11)
O72X—Mg4—O84X^xix^	172.1 (5)	O11^xxiii^—Na5—O33^xxix^	147.69 (19)
O72X^iii^—Mg4—O84X^xix^	163.0 (7)	O31—Na5—O33^xxix^	100.52 (15)
O33^xxi^—Mg4—O84X^xxiii^	87.6 (2)	O31^vii^—Na5—O33^xxix^	53.08 (11)
O33^xxii^—Mg4—O84X^xxiii^	102.3 (2)	O33^ii^—Na5—O33^xxix^	60.72 (15)
O41^xxi^—Mg4—O84X^xxiii^	71.3 (3)	O11^xvi^—Na5—O61	62.08 (13)
O41^xxii^—Mg4—O84X^xxiii^	85.9 (3)	O11^xxiii^—Na5—O61	99.82 (18)
O72X—Mg4—O84X^xxiii^	163.0 (7)	O31—Na5—O61	98.93 (17)
O72X^iii^—Mg4—O84X^xxiii^	172.1 (5)	O31^vii^—Na5—O61	59.67 (13)
O84X^xix^—Mg4—O84X^xxiii^	20.3 (4)	O33^ii^—Na5—O61	144.13 (18)
O61^xxiv^—Mg5—O11^vii^	87.23 (15)	O33^xxix^—Na5—O61	112.32 (12)
O61^xxiv^—Mg5—O53^viii^	81.09 (15)	O11^xvi^—Na5—O61^vii^	99.82 (18)
O11^vii^—Mg5—O53^viii^	105.84 (15)	O11^xxiii^—Na5—O61^vii^	62.08 (13)
O61^xxiv^—Mg5—O42^ix^	86.34 (19)	O31—Na5—O61^vii^	59.67 (13)
O11^vii^—Mg5—O42^ix^	166.46 (18)	O31^vii^—Na5—O61^vii^	98.93 (17)
O53^viii^—Mg5—O42^ix^	84.92 (16)	O33^ii^—Na5—O61^vii^	112.32 (12)
O61^xxiv^—Mg5—O23	174.75 (16)	O33^xxix^—Na5—O61^vii^	144.13 (18)
O11^vii^—Mg5—O23	87.55 (13)	O61—Na5—O61^vii^	50.43 (16)
O53^viii^—Mg5—O23	100.94 (14)	O11^xvi^—Na5—O13^xxiii^	102.48 (15)
O42^ix^—Mg5—O23	98.63 (18)	O11^xxiii^—Na5—O13^xxiii^	53.73 (12)
O61^xxiv^—Mg5—O24	111.86 (16)	O31—Na5—O13^xxiii^	90.11 (11)
O11^vii^—Mg5—O24	84.91 (13)	O31^vii^—Na5—O13^xxiii^	146.76 (19)
O53^viii^—Mg5—O24	163.94 (15)	O33^ii^—Na5—O13^xxiii^	62.13 (12)
O42^ix^—Mg5—O24	86.44 (15)	O33^xxix^—Na5—O13^xxiii^	94.41 (16)
O23—Mg5—O24	67.04 (12)	O61—Na5—O13^xxiii^	149.41 (17)
O74X—Mg6—O13^xxv^	89.1 (2)	O61^vii^—Na5—O13^xxiii^	113.70 (11)
O74X—Mg6—O83X	56.3 (3)	O11^xvi^—Na5—O13^xvi^	53.73 (12)
O13^xxv^—Mg6—O83X	92.0 (2)	O11^xxiii^—Na5—O13^xvi^	102.48 (15)
O74X—Mg6—O44^xxii^	86.4 (2)	O31—Na5—O13^xvi^	146.76 (19)
O13^xxv^—Mg6—O44^xxii^	174.7 (2)	O31^vii^—Na5—O13^xvi^	90.11 (11)
O83X—Mg6—O44^xxii^	87.8 (2)	O33^ii^—Na5—O13^xvi^	94.41 (16)
O74X—Mg6—O33^xxii^	128.5 (3)	O33^xxix^—Na5—O13^xvi^	62.13 (12)
O13^xxv^—Mg6—O33^xxii^	91.79 (15)	O61—Na5—O13^xvi^	113.70 (11)
O83X—Mg6—O33^xxii^	173.9 (2)	O61^vii^—Na5—O13^xvi^	149.41 (17)
O44^xxii^—Mg6—O33^xxii^	88.92 (16)	O13^xxiii^—Na5—O13^xvi^	65.17 (14)
O74X—Mg6—O34^xxii^	164.9 (3)	Na6^xxx^—Na6—O53^vii^	112.87 (15)
O13^xxv^—Mg6—O34^xxii^	88.80 (15)	Na6^xxx^—Na6—O71^xi^	82.19 (13)
O83X—Mg6—O34^xxii^	108.8 (2)	O53^vii^—Na6—O71^xi^	149.5 (3)
O44^xxii^—Mg6—O34^xxii^	96.32 (17)	Na6^xxx^—Na6—O42^xxxi^	147.02 (18)
O33^xxii^—Mg6—O34^xxii^	66.49 (13)	O53^vii^—Na6—O42^xxxi^	67.95 (17)
O74X—Mg6—O82X	99.5 (3)	O71^xi^—Na6—O42^xxxi^	114.2 (2)
O13^xxv^—Mg6—O82X	98.5 (2)	Na6^xxx^—Na6—O53^xi^	54.33 (13)
O83X—Mg6—O82X	43.6 (2)	O53^vii^—Na6—O53^xi^	58.54 (18)
O44^xxii^—Mg6—O82X	85.0 (2)	O71^xi^—Na6—O53^xi^	129.4 (3)
O33^xxii^—Mg6—O82X	131.0 (2)	O42^xxxi^—Na6—O53^xi^	116.5 (2)
O34^xxii^—Mg6—O82X	66.03 (19)	Na6^xxx^—Na6—O21	138.10 (16)
O74X—Mg6—O72X^iii^	57.0 (3)	O53^vii^—Na6—O21	66.58 (18)
O13^xxv^—Mg6—O72X^iii^	96.6 (4)	O71^xi^—Na6—O21	84.4 (2)
O83X—Mg6—O72X^iii^	112.4 (3)	O42^xxxi^—Na6—O21	74.1 (2)
O44^xxii^—Mg6—O72X^iii^	78.6 (4)	O53^xi^—Na6—O21	109.8 (2)
O33^xxii^—Mg6—O72X^iii^	71.9 (2)		

Symmetry codes: (i) −*x*, *y*, *z*−1; (ii) *x*, *y*, *z*−1; (iii) −*x*, *y*, *z*; (iv) −*x*, −*y*+1, *z*−1/2; (v) *x*, −*y*+1, *z*−1/2; (vi) *x*−1/2, *y*−1/2, *z*; (vii) −*x*+1, *y*, *z*; (viii) *x*+1/2, *y*−1/2, *z*; (ix) −*x*+1, −*y*+1, *z*−1/2; (x) −*x*+1/2, −*y*+1/2, *z*−1/2; (xi) *x*+1, *y*, *z*; (xii) *x*, *y*, *z*+1; (xiii) −*x*+1/2, −*y*+3/2, *z*+1/2; (xiv) −*x*, −*y*+1, *z*+1/2; (xv) −*x*+1, −*y*+1, *z*+1/2; (xvi) *x*+1/2, *y*+1/2, *z*; (xvii) *x*−1/2, −*y*+3/2, *z*+1/2; (xviii) *x*−1, *y*, *z*; (xix) *x*−1/2, *y*+1/2, *z*; (xx) −*x*+3/2, *y*+1/2, *z*; (xxi) *x*−1/2, −*y*+3/2, *z*−1/2; (xxii) −*x*+1/2, −*y*+3/2, *z*−1/2; (xxiii) −*x*+1/2, *y*+1/2, *z*; (xxiv) −*x*+3/2, *y*−1/2, *z*; (xxv) *x*, −*y*+1, *z*+1/2; (xxvi) −*x*+1/2, *y*−1/2, *z*; (xxvii) −*x*+1/2, −*y*+1/2, *z*+1/2; (xxviii) *x*−1/2, −*y*+1/2, *z*+1/2; (xxix) −*x*+1, *y*, *z*−1; (xxx) −*x*+2, *y*, *z*; (xxxi) *x*+1/2, −*y*+3/2, *z*−1/2.
